# Dimensions of well-being and social harmony of older persons in Ghana: A secondary analysis of longitudinal survey data of the 2014/15 Study on Global Ageing and Adult Health (SAGE Wave 2)

**DOI:** 10.1371/journal.pone.0314666

**Published:** 2024-12-31

**Authors:** Joseph Kojo Oduro, Akwasi Kumi-Kyereme

**Affiliations:** Department of Population and Health, College of Humanities and Legal Studies, University of Cape Coast, Cape Coast, Ghana; King Khalid University, EGYPT

## Abstract

**Objectives:**

A crucial factor in healthy ageing is age-friendly environments for older persons. The opportunities that come with getting older as well as the safety of older persons are influenced by their social surroundings and overall well-being, yet, there is a dearth of research focus on this subject. This study examined the association between dimensions of well-being and the social harmony of older persons in Ghana.

**Methods:**

A secondary analysis of longitudinal survey data of the 2014/15 Study on Global Ageing and Adult Health (SAGE Wave 2) conducted by the World Health Organization was used. Only older adults 60 years and older were included in this study. Multilevel logistic regression techniques were used to examine dimensions of well-being as predictors of social harmony of older persons. The output was reported as odds ratios (OR).

**Results:**

Results show that high levels of emotional and physical well-being were 13.5% and 14.3% more likely to be associated with a high level of social harmony (OR = 1.35, 95% CI = 1.35,1.35), (OR = 1.43, 95% CI = 1.43,1.43). However, older persons with high levels of psychological and spiritual well-being were 7% and 3% less likely to experience a high level of social harmony (OR = 0.73, 95% CI = 0.63,0.93), (OR = 0.39, 95% CI = 0.39,0.40).

**Conclusion:**

This study shows a multifarious association between dimensions of well-being and the social harmony of older persons. A positive association is found between high levels of emotional and physical well-being and social harmony in older persons. However, older persons with high levels of psychological and spiritual well-being showed less experience of social harmony. This has implications for policy for improving older persons’ well-being and social harmony. Policies and social interventions should consider the various needs and situations of older persons to establish an environment of safety and opportunities concerning higher social harmony in Ghanaian society.

## Introduction

A crucial factor in healthy ageing is age-friendly environments for older persons [1, 2]. The opportunities that come with getting older as well as the safety of older persons about crime and violence are greatly influenced by their surroundings and overall well-being, yet, there is a dearth of research focus on this subject [1]. Globally, Africa is leading in terms of the fastest-growing population of older persons (i.e., persons aged 60 years and older), with Latin America, the Caribbean, and Asia following suit [3]. The share of the population that is 60 years of age or older is growing at an alarming rate [4]. Globally, there were one billion individuals 60 years or older in 2019 [4, 5]. By 2030, there will be 1.4 billion, and by 2050, there will be 2.1 billion of this cohort [5–7]. This increase is happening at a never-before-seen rate, and it is going to accelerate quickly in the upcoming decades, especially in developing nations. Like Zimbabwe, Namibia, India, and Indonesia, Ghana is projected to witness a substantial surge in its population of older persons aged 60 years and above to approximately 11.9% by 2050, but current data indicates that 13.1% of the population already falls within this age bracket [8, 9].

Essentially, research on the social harmony of older persons in communities must increasingly become the centre of focus in the study of gerontology, especially in a global setting where the number of people 60 years of age and older surpasses that of children under 5 and youth aged 15 to 24 years [10]. High levels of social harmony are defined as the safety of older persons in their homes, on the streets after dark, and in the absence of crime or violence [11]. A safe and or secure environment for the older population is an important prospect for high social harmony. It also improves the quality and age-friendly environment for older persons [12]. Notwithstanding its important role, there is a dearth of evidence on the factors influencing older persons’ social harmony.

Several studies have provided a prised understanding of the experiences and difficulties confronting older persons in preserving social harmony [13–16]. These studies underline the importance of social inclusion, dignity in care, self-management, and social participation in enhancing the overall well-being of older persons [14]. Evidence shows that social support, cognitive reappraisal, and social comparison impact the social harmony of older persons [17]. Also, emotionally intelligent older persons had higher levels of perceived support from family and friends, resulting in higher social harmony indicators [18]. Physical activity impacts the physical well-being of older persons and contributes to their overall well-being and social harmony [19]. Similarly, physical well-being enhances feelings of belonging and social cohesion which is linked to social harmony [19]. There is a positive association between psychological well-being and social harmony in older persons [19, 20]. Well-being is positively predicted by spirituality experienced through connectedness with the transcendent and through connectedness with others [20].

Studies show that the quality of life of older residents in Ghana’s slum communities is negatively impacted by several factors, including inadequate access to social and healthcare services, spiritual beliefs that shape their behaviour while seeking medical attention, and unfulfilled social demands [21, 22]. Ghana’s social changes are distancing older persons which adversely affects their social harmony. Urbanisation, migration, and demands on education, and employment, make it difficult for many families to care for their older relatives [23, 24]. Older persons often provide long-term care for grandchildren and other relatives while frequently sharing a home with other family members [25]. Persistent loneliness in older persons can result in depression and other mental health issues [19]. In Ghana, there is a mismatch between the supply and demand for institutional support networks for older persons [26]. Social fragility is linked to greater rates of depression in the older population, particularly in widowed or divorced individuals [27]. Some older persons in Ghana continue to work after age 60, either for the government or private sector [25]. While these studies focused on very important concerns about older persons, they do not address the aspects of well-being that could influence the contemporary age-friendly environmental-related issues concerning their social harmony.

Thus, there is a notable lack of studies examining the aspects of well-being and high social harmony of older persons in the Ghanaian context, despite the crucial role that social harmony plays in the lives of older persons. This lack of evidence impedes not only our ability to comprehend the underlying factors of older persons’ social harmony but also the ability to improve age-friendly environments for older persons. This study examined the association between dimensions of well-being, including emotional, physical, psychological, and spiritual well-being, and the social harmony of older persons in Ghana. The study focused on factors associated with high social harmony, aiming to enhance existing social interventions to improve the age-friendly environment of older persons. It argues that while many policies aim to improve less favourable situations, there is a need to also focus on the achievements made from existing social interventions. Therefore, this study uniquely concentrates on high social harmony rather than low social harmony of older persons.

## Materials and methods

This study used the comprehensive pooled data on the health and well-being of older persons in Ghana from the World Health Organisation 2014/15 Ghana Study on Global Ageing and Adult Health (SAGE) Survey Wave 2. SAGE Ghana is a longitudinal survey programme coordinated by the WHO’s multi-country studies unit and is a nationally representative study undertaken in six countries, including China, Ghana, India, Mexico, the Russian Federation and South Africa. The SAGE programme seeks to strengthen, gather, process, and manage data to understand the needs and challenges of older persons to inform policy, planning and research (US National Institute on Aging [NIA] & WHO, 2014). SAGE Ghana is scheduled to collect data every four years up until 2018/19. The 2014/15 (Wave 2) SAGE collected some information on the well-being of older persons, including their emotional, physical, psychological, spiritual well-being, and social harmony, in addition to socio-demographic and economic data. Even though SAGE data is collected every four years only the Wave 2 2014/15 was in the public domain at the time this study was conducted. Thus, the Wave 2 was used.

SAGE surveys follow the standard procedures (i.e., sampling, questionnaire development, data collection, cleaning, coding, and analysis) which allow for cross-country comparison. The survey employs a stratified two-stage sampling technique. The initial stage involved stratification of the country by region and urban-rural location. Primary Sampling Units (PSUs), also referred to as Census Enumeration Areas from the 2010 Ghana Population and Housing Census, were allocated proportionately to the number of PSUs in each region by rural-urban location. PSUs were selected independently within each stratum and 214 PSUs were sampled. Households were selected in each PSU in the second stage using a systematic random sampling approach. All members aged 50 years and above were selected for interviews in the selected households. Overall, 3,575 respondents aged 50 years and above were interviewed. For the present study, only older persons 60 years and above with complete data were included in the analysis. Thus, older respondents below 60 years were excluded. Respondents were categorised according to functional age brackets: 60–69 (young-old), 70–79 years (old-old) and 80+ years (oldest old) [28–30] for the analysis.

The methods in this study were conducted in line with the relevant guidelines and regulations conforming to the Belmont and Helsinki Declarations. The Wave 2 of the WHO-Ghana SAGE was approved by the Ethics Review Committee, World Health Organisation, Geneva, Switzerland, and the Ghana Health Service. Written informed consent was given to individual respondents. However, the author of this manuscript was not directly involved in the data collection processes but rather obtained access by requesting for the data. Thus, ethical approval was not obtained for this study.

### Study variables

#### Outcome variables

Social harmony of the older persons was the outcome variable of interest. The outcome variable was measured by three variables as were used in the Ghana 2014/15 SAGE. It was measured in SAGE as "safety in the area where older persons live”. In this study, social harmony was conceptualised as the state of safety and peaceful interactions with the environment among older persons in a community or society. It is typified by a feeling of security, free from fear of injury, and collaboration amongst members of society. Given that the social harmony of older persons is a latent indicator, SAGE Ghana Wave 2 developed a series of questions to measure it, including, “In general,…….1) how safe from crime and violence do you feel when you are alone at home?” 2 How safe do you feel when walking down your street alone after dark?”, and 3)“. . . have you or anyone in your household been the victim of a violent crime, such as assault or mugging?”. The first two questions were rated on a 5-point Likert scale to reflect their self-rating, ranging from 1 = Completely safe, 2 = Very safe, 3 = Moderately safe, 4 = Slightly safe, to 5 = Not safe at all. Response categories for the last question were dichotomous, 1 = Yes, and 2 = No.

To examine the predictors of the outcome variable of older persons, dummies were created and factor analysis was used to derive an indicator for social harmony. The factor analysis technique was used to create a composite variable to measure the overall social harmony of older persons. This technique was adopted because it circumvents the problem of multicollinearity and assigns indicator weights based on the variations in the responses. The mathematical procedure in FA transforms correlated variables into fewer uncorrelated variables (factor scores). The factor scores are a linear combination of the original variables which are derived in decreasing order of importance with the first factor score accounting for as much of the variability in the data as possible, and each succeeding factor accounting for as much of the remaining variability.

The factor loadings for all the indicators are indicative of positive social harmony, suggesting that the first-factor score represents the overall social harmony of older persons. Table 1 shows similar factor loadings for all the variables measuring social harmony, indicating that all the indicators contribute almost equally to the overall social harmony of older persons. To identify the factors associated with high social harmony, the first factor scores were ranked and the top 20.0% coded as “1” to represent those with high social harmony, while “0” represents low social harmony. Thus, the study examined the variables linked to older persons’ social harmony. Details in Table 1.

**Table 1:** Social harmony, indicators, and factor loadings.

Domain	Indicator	Factor loading
Social harmony	1. Feel safe at home from crimes	0.944
	2. Feel safe on the street alone after dark	0.930
	3. Not been victim of a crime or violence	0.119

#### Independent variables

The key independent variable was the dimensions of well-being. In this study, the dimensions of well-being referred to the optimal experience and functioning of older persons through high emotional, physical, psychological and spiritual well-being. In the SAGE wave 2, emotional well-being was measured as, in the last 30 days, how much of a problem did you have…1) …with feeling sad, low or depressed? 2) … with worry or anxiety? 3)..with feeling lonely? And 4)…with feeling neglected. Physical well-being was measured using the functioning assessment scale. Questions used were, in the last 30 days, how much difficulty did you have …1) … in bathing/washing your whole body? 2).. … with eating (including cutting up your food)? and 3) … in vigorous activities (‘vigorous activities’ require hard physical effort and cause large increases in breathing or heart rate? Psychological well-being was measured as, “Overall in the last 30 days, how much difficulty…1)…did you have with concentrating or remembering things? Each of the above questions was rated on a 5-point Likert scale to reflect their self-rating, ranging from 1 = None, 2 = Mild, 3 = Moderate, 4 = Severe to 5 = Extreme/Cannot do. 2) How often have you found that you could not cope with all the things that you had to do? 3) How often have you felt that you were unable to control the important things in your life? Each of these questions was rated on a 5-point Likert scale ranging from 1 = never, 2 = Rarely, 3 = Sometimes, 4 = Fairly often to 5 = Very often. Lastly, spiritual well-being was measured by 1) “Do you belong to a religious denomination? Responses were 1 = No, none, 2 = Buddhism, 3 = Chinese traditional religion, 4 = Christianity (including roman catholic, protestant, orthodox, other), 5 = Hinduism, 6 = Others. 2)… attended religious services (not including weddings and funerals). This was also rated on a 5-point Likert scale to reflect their self-rating, ranging from 1 = never, 2 = once or twice per year, 3 = once or twice per month, 4 = once or twice per week to 5 = daily.

Factors analysis was used to create an indicator of the emotional, physical, psychological, and spiritual well-being of older persons. The first-factor score for each of the dimensions of well-being was used to represent the specific variable of well-being. Details are in Table 2.

**Table 2:** Dimensions of well-being, indicators, and factor loadings.

Domain	Indicator	Factor loadings
Emotional wellbeing	Not depressed	0.813
	Not worried	0.761
	Do not feel lonely	0.794
	Do not feel neglected	0.774
Physical wellbeing	Able to bath/wash whole body	0.848
	Able to eat and cut up food	0.816
	Able to do moderate/vigorous exercise	0.704
Psychological wellbeing	Able remember things	0.924
	Able to concentrate on things I do	0.907
	Able to cope in difficult times	0.850
	Have control over what I do	0.741
Spiritual wellbeing	Belong to a religion/church	0.929
	Participate in religious services	0.691

### Confounders

The confounders selected for the study included sex, age, marital status, education, ethnicity, residence, ecological zones, perceived health, work status, and income. These confounders were chosen based on evidence of their potential influence on the relationship between dimensions of well-being and social harmony [31–34]. In the Ghanaian context, controlling for these factors is crucial due to their important impact on social harmony. By accounting for these confounders, the study findings, allow for a more accurate understanding of the specific relationship between dimensions of well-being and social harmony in Ghana. Details are presented in Table 3.

**Table 3:** Confounding factors.

Variables	Measurement
Sex	0 = Male
	1 = Female
Age	0 = 60–69 [young old]
	1 = 70–79 [old old]
	2 = 80+ [oldest old]
Marital status	0 = Not married
	1 = Married
	2 = Cohabiting/separate/divorced
	3 = Widowed
Educational level	0 = No formal education
	1 = Primary/JHS
	2 = Secondary/Higher
Ethnicity	0 = Akan
	1 = Ewe
	2 = Ga-Adangbe
	3 = Mande-Busanga
	4 = Others
Place of residence	0 = Urban
	1 = Rural
Ecological zones	0 = Savannah
	1 = Forest
	2 = Coastal
Perceived general health	0 = Very bad
	1 = Moderate
	2 = Very good
Work status	0 = Not working
	1 = Full time
	2 = Part time
Income	0 = Low income
	1 = High income

### Statistical analysis

Data was analysed using SPSS (version 26.0) and R studio (version 4.3.1). First, descriptive statistics were performed to describe older persons and their background characteristics. Second, cross-tabulations with chi-squared tests were used to examine the percentage distribution of the older persons with high social harmony by the dimensions of well-being and the confounders. Lastly, multilevel binary logistic regression was used to examine the dimensions of well-being and confounders that were significantly (p < 0.05) associated with the high social harmony of older persons.

#### Multilevel binary logistic regression

Multilevel binary logistic regression was conducted to examine the dimensions of well-being associated with the probability of an older person having high social harmony, accounting for the confounders and the level of nesting of respondents at the community level. The approach prevented the possibility of underestimation or overestimation of model parameters due to the stratified nature of SAGE Ghana Wave 2 [35]. A two-level multilevel binary logistic regression model was fitted since the outcome variable of interest was dichotomous coded “1” if an older respondent had high social harmony and “0” if otherwise and with individuals nested within communities. The implication of a statistically significant (p < 0.05) random variance at the community level is that the community in which the respondent resides influences his/her social harmony. All the dimensions of well-being were retained in the model irrespective of their significance level, whilst only statistically significant confounders were retained in the model as suggested by Hao et al., [36].

#### Test for multicollinearity

To ensure that the model fitted was stable, a test of multicollinearity was conducted. The interval-by-interval Pearson’s R and the ordinal-by-ordinal Spearman correlation were used to examine the extent of correlations among categorical-by-categorical covariates. The results showed very low correlations among the variables, thus, low potential for multicollinearity.

#### Model fitting procedure

A sequential modelling approach was used to examine the association between social harmony and dimensions of well-being, accounting for the important background characteristics. Model 1 included the place of residence to account for some of the survey weights. Model 2 added the dimensions of well-being, while Model 3 included the background characteristics. A two-level (individuals nested within communities) multilevel binary logistic regression was used to examine the predictors of high social harmony among older persons. Interpretation of all the model results was based on the final models (Table 4, Model 3).

**Table 4:** Social harmony by dimensions of well-being.

Community variable	Model I	Model 2	Model 3
	OR [95% CI]	OR [95% CI]	OR [95% CI]
Place of residence			
Urban	1.00	1.00	1.00
Rural	1.02[0.91,1.52]	1.54[0.87,2.76]	1.35[0.95,2.25]**
*Dimensions of wellbeing*			
Emotional Wellbeing			
Low		1.00	1.00
High		1.47[1.05,2.04]*	1.35[1.35,1.35]**
Physical Wellbeing			
Low		1.00	1.00
High		1.43[1.03,1.98]*	1.43[1.43,1.43]**
Psychological Wellbeing			
Low		1.00	1.00
High		0.79[0.57,1.08]	0.73[0.63,0.93]**
Spiritual Wellbeing			
Low		1.00	1.00
High		0.41[0.30,0.57]**	0.39[0.39,0.40]*
*Confounding Factors*			
Sex			
Male			1.00
Female			0.68[0.67,0.88]**
Education			
No formal education			1.00
Primary/JHS			0.64[0.54,0.96]**
Secondary/Higher			1.01[0.98,1.22]**
Marital status			
Never married			1.00
Married			1.21[1.01,2.25]**
Separated/divorced			1.28[1.27,2.48]**
Widowed			1.32[0.93,2.35]*
Work status			
Not working			1.00
Full time			1.47[1.03,2.43]**
Part time			2.11[1.96,3.48]**
General health			
Poor			1.00
Moderate			0.51[0.50, 0.75]
Good			0.45[0.32,0.68]**
Income			
Low			1.00
High			0.86[0.76,0.99]**
Variance of the random Effects [SE]			
Community	3.447[0.03]**	3.414[0.03]**	3.596 [0.03] **
% Δ in random effect	+2.83	+2.46	+2.36
Community			
Deviance	2068.3	2066.2	2018.3
AIC	2080.3	2080.2	2054.3

The intraclass correlation coefficient (ICC) 2,1 was used to estimate the amount of variation in social harmony of older persons that is attributable to differences in the community. ICC values <0.5 (poor variation), between 0.5–0.75 (moderate), between 0.75–0.9 (good), and >0.90 (excellent) [37].

The Akaike Information Criterion (AIC) was used to determine the model of best fit (AIC). This AIC is used to select the model that reduces the negative likelihood relative to the number of parameters in the model. Specifically, the model with the least expected information loss [38–40]. Thus, the lower the AIC, the better the model and the closer it is to the unidentified population model.

## Results

### Background characteristics of respondents

Out of the 3, 575 older persons, 1,927 fulfilled the inclusion criteria and were included in the analysis (Mean±SD 14.1±62.6 years; 43.2% male; 56.8% female). Apart from physical well-being which was low (69.4%), respondents had high emotional (61.9%), psychological (54.9%), spiritual (80.2%) well-being, and high social harmony (67.3%). Complete information on respondent background characteristics is provided in Table 5.

**Table 5:** Background characteristics of respondents (n = 1927).

Background characteristics	Sample size	Percent (%)
Sex		
Male	833	43.2
Female	1094	56.8
Age		
60–69	652	33.8
70–79	623	32.3
80+	652	33.8
Mean±SD		14.1±62.6
Marital status		
Not married	122	6.3
Married	1126	58.5
Separated/divorced	207	10.7
Widowed	472	24.5
Education		
No formal education	867	45.0
Primary or JHS	523	27.1
Secondary or higher	537	27.9
Religion		
Christianity	1402	72.8
Islam	355	18.4
No religion/Indigenous	170	8.8
Ethnicity		
Akan	886	46.0
Ewe	118	6.1
Ga-Adangbe	290	15.1
Mande-Busanga	454	23.6
Others	178	9.2
Work status		
Not working	372	19.3
Part time work	642	33.3
Full time work	913	47.4
Ecological Zones		
Savannah	418	21.7
Forest	956	49.6
Coastal	553	28.7
Place of residence		
Urban	753	39.1
Rural	1174	60.9
Income		
Low	448	23.3
High	1479	76.7
General health		
Poor	168	8.7
Moderate	444	23.0
Good	1315	68.2
*Dimensions of Wellbeing*		
Emotional wellbeing		
Low	735	38.1
High	1192	61.9
Physical wellbeing		
Low	1337	69.4
High	590	30.6
Psychological wellbeing		
Low	870	45.1
High	1057	54.9
Spiritual wellbeing		
Low	382	19.8
High	1545	80.2
Social harmony		
Low	631	32.7
High	1296	67.3

### Social harmony, background characteristics and dimensions of well-being

High levels of social harmony differed by the demographic characteristics of older persons. For instance, 47% of the males and 41% of the oldest old (80+) had a high level of social harmony. Also, 44.1% with secondary/higher education and 46% who worked full-time had a high level of social harmony. Regarding the dimensions of well-being, respondents who had high levels of emotional (42.7%), physical (43.9%), psychological (44.0%), and spiritual (64.1%) well-being had high level of social harmony. Complete details of the results are in Table 6.

**Table 6:** Social harmony, demographics and dimensions of well-being (n = 1927).

	Social Harmony	
*Background characteristics*	High	*χ* ^2^
	%	
Sex		27.137**
Male	47.4	
Female	35.6	
Age		0.169
60–69 years	40.6	
70–79 years	40.1	
80 years or older	41.3	
Marital status		2.936
Never married	43.4	
Married	42.0	
Cohabiting/Separated/divorced	38.8	
Widowed	37.9	
Education		14.216**
No formal education	42.7	
Primary/JHS	33.8	
Secondary/higher	44.1	
Religion		12.732**
Christianity	39.3	
Islam	40.0	
No religion/Indigenous	53.5	
Ecological Zones		3.928
Savannah	37.6	
Forest	40.3	
Coastal	43.8	
Place of residence		2.607
Urban	38.5	
Rural	42.2	
Ethnicity		11.603*
Akan	41.4	
Ewe	50.0	
Ga-Adangbe	42.1	
Mande-Busanga	34.9	
Others	43.8	
Main occupation		22.417**
Not working	32.6	
Part time	38.0	
Full time	46.0	
Income		0.806
Low	42.5	
High	40.2	
General health		6.959*
Poor	48.2	
Moderate	36.7	
Good	41.1	
*Dimensions of Wellbeing*		*χ* ^*2*^
Emotional wellbeing		1.906
Low	39.5	
High	42.7	
Physical wellbeing		18.232**
Low	33.6	
High	43.9	
Psychological wellbeing		7.134**
Low	38.0	
High	44.0	
Spiritual wellbeing		108.380**
Low	34.9	
High	64.1	

χ2 = Chi square test

**p<0.01

*p<0.05

### Regression analysis of high social harmony and the dimensions of well-being

Place of residence, the dimensions of well-being (emotional, physical, psychological, and spiritual well-being), and all the confounding factors were significant predictors of high social harmony of older persons 60 years and older. When the place of residence was introduced in Model 1, the variance of the random effects at the community level increased by 2.83% and remained statistically significant at p < 0.05. This shows that significant differences exist between communities about older persons with high social harmony, after accounting for the place of residence. When the dimensions of well-being were included in model 2, the variance of the random effects at the community level reduced marginally by 2.46%, but the effect remained statistically significant at p < 0.05. Further indicating that significant differences exist between communities concerning high social harmony among older persons, even after accounting for the place of residence and the dimensions of well-being. When the background characteristics were included in Model 3, the variance of the random effects at the community level marginally reduced by 2.36% and remained statistically significant at p < 0.05. This further indicates that the background factors increase the variability between communities. The statistically significant variance of the random effects at the community level after accounting for all the selected variables in the model indicates that the selected variables do not explain all the differences between communities about older persons with high social harmony.

After controlling for important predictors in the model, the results indicate that older persons in the rural communities had higher odds (OR = 1.35, 95% CI = 0.95,2.25) of having high social harmony when compared with those in urban areas. Concerning the dimensions of well-being, older persons who had high levels of emotional and physical well-being were 13.5% and 14.3% more likely to be associated with a high level of social harmony (OR = 1.35, 95% CI = 1.35,1.35), (OR = 1.43, 95% CI = 1.43,1.43) when compared with those who had low levels of emotional and physical well-being. However, older persons with high levels of psychological and spiritual well-being were 7% and 3% less likely to experience a high level of social harmony (OR = 0.73, 95% CI = 0.63,0.93), (OR = 0.39, 95% CI = 0.39,0.40) when compared with those with a low level of psychological and spiritual well-being.

Being a female was 6% less likely to be associated with a high level of social harmony (OR = 0.68, 95% CI = 0.67,0.88) compared with being a male. Having a secondary/higher education was 10% more likely to be associated with high social harmony (OR = 1.01, 95% CI = 0.98,1.22), compared to those with no former education. Also, older persons who were ever married (married, separated/divorced, widowed) (OR = 1.21, 95% CI = 1.01,2.25), (OR = 1.28, 95% CI = 1.27,2.48), and (OR = 1.32, 95% CI = 0.93,2.35) respectively, had higher odds of high social harmony when compared with older persons who were never married. Furthermore, older persons who engaged in full-time work were 21% more likely to be associated with a high level of social harmony (OR = 2.11, 95% CI = 1.96,3.48), compared to those who were not working. Surprisingly, having a good perceived health status was 4% less likely to be associated with a high level of social harmony (OR = 0.45, 95% CI = 0.32,0.68) when compared with having a poor perceived health status.

## Discussion

Key findings of this study are that older persons with high levels of emotional and physical well-being were more likely to be associated with a high level of social harmony when compared with those with low levels of emotional and physical well-being. However, older persons with high levels of psychological and spiritual well-being were less likely to experience a high level of social harmony when compared with those with a low level of psychological and spiritual well-being.

Older persons with a high level of emotional well-being were associated with a high level of social harmony. This is likely due to the positive effects of Ghanaian social support and strong social connections which improve the social harmony of older persons. Evidence shows that social support, cognitive reappraisal, and social comparison impact the social harmony of older persons [17]. Also, emotionally smart older persons had higher levels of perceived support from family and friends, resulting in higher social harmony indicators. Further, emotionally gratifying relationships and social and emotional support provide a buffer for older persons against the challenges inherent in the ageing process [18].

The finding shows a positive association between a high level of physical well-being and a high level of social harmony among older persons. This is consistent with a similar study which shows a positive relationship between different forms of physical activities and physical well-being [41]. This suggests that physical activity impacts the physical well-being of older persons which may contribute to their overall well-being and social harmony. Similarly, physical well-being was found to enhance feelings of belonging and social cohesion which is associated with social harmony of older persons [42].

However, a high level of psychological well-being was less likely associated with a high level of social harmony. In contrast, existing studies in general, suggest a positive association between psychological well-being and social harmony in older persons [43, 44]. It is important to note that the association between psychological well-being and social harmony can be influenced by factors such as individual differences in older persons, cultural context (i.e., *values*, *beliefs etc*.), and the specific measures of well-being and social harmony in the study [45]. More research is needed to fully understand this relationship, especially in the context of older persons in Ghana.

Also, older persons with a high level of spiritual well-being were less likely to experience a high level of social harmony. This finding appears counterintuitive given that a high level of spiritual well-being is mostly associated with positive social outcomes. Evidence shows that spirituality has a direct effect on the mental quality of life among older persons [46]. Indicating that a high level of spiritual well-being could potentially influence an older person’s perception of social harmony [47]. However, a plausible reason could be that older persons with high levels of spiritual well-being might be more focused on their personal spiritual growth and less on their social harmony.

Older persons in rural communities had the higher experience of social harmony when compared with those in urban areas. Some older persons in rural areas of Ghana may perceive a higher level of social harmony due to factors such as safety, faith in God, support from family and friends, and trustworthiness [31, 48]. On the other hand, on account of urbanisation, socioeconomic development and globalisation, the traditional system of respect, protection and care for older persons is breaking down [48]. Thus, considering the needs and resources available to older persons in rural areas, their colleagues in urban slums may perceive social harmony differently in Ghana.

Being a female was less likely to be associated with a high level of social harmony compared to being a male. This could be due to variations in the social needs of older men and women in Ghana. Satisfying social needs is important for older persons to stay healthy and community-dwelling. This implies that social harmony could be influenced by how well these social needs are met, which could vary between older males and females [23, 27]. Older persons in Ghana, especially older women provide long-term care for grandchildren and other relatives and this could lead to less experience of social harmony [9, 13].

Having a secondary/higher education was more likely to be associated with high social harmony, compared to those with no former education. This contradicts that among older adults, engagement in education can potentially have positive effects on cognition and psychological well-being and can prevent social isolation [24]. Suggesting that social harmony could be influenced by the level of education, as education often plays a crucial role in shaping individuals’ social interactions and perceptions of social harmony. That older adults are capable, motivated, and active learners [31]. The educational programs they engaged in were driven by the life context of older adulthood [32]. This suggests that older adults with a formal education might experience more social harmony than those with no formal education.

Also, older persons who were ever married (i.e., *married*, *separated/divorced*, *widowed*) had higher odds of high social harmony when compared with older persons who were never married. The results showed that widows had higher levels of informal social participation than non-widowed persons, whereas formal social participation levels were comparable between the two groups [32, 49]. This suggests that being ever married could potentially influence social harmony among older persons 60 years and older.

Furthermore, older persons who engaged in full-time work were more likely to be associated with a high level of social harmony, compared to those who were not working. This could plausibly be because some older persons in Ghana continue to work after age 60, either for the government or private sector [9]. This corroborates with the evidence that age, social identity, and work outcomes are interconnected [49]. Suggesting that individuals psychologically internalize their social group memberships, and these social identities influence their cognition, affect, motivation, and behaviour in important ways. The implication is that older persons who are engaged in full-time work might have a stronger social identity, which could potentially influence their level of social harmony. Workplace interventions that support older employees’ health and workability can have a positive impact on their well-being [50]. This suggests that older adults who are engaged in full-time work might benefit from such interventions, which could potentially enhance their level of social harmony.

Surprisingly, having a good perceived health status was less likely to be associated with a high level of social harmony among older persons. This suggests that perceptions of an older person influence their social harmony. Evidence reveals that intrapersonal factors such as being older and having good health status are positively associated with satisfaction with the healthcare system which impacts the level of social harmony [51].

Generally, there are limited studies in Ghana on sociodemographic and social harmony. however, existing evidence shows that Ghana’s social changes are distancing older persons which adversely affects their social harmony. The issue of urbanisation, migration, and demands on education, and employment, make it difficult for many families to care for their older relatives [31]. Persistent loneliness in older persons can result in depression and other mental health issues which impact their social harmony [52].

### Strengths and limitations

One strength of this study was that only variables that showed significant association with social harmony were included in the multilevel logistic regression analysis. This supports the robustness of the models and reinforces the reliability and replicability of the findings of this study. Also, a large and national representative of the data sets from the SAGE 2014/15 Wave 2 was used in the study. The study offers valuable insights into the impact of the dimensions of well-being on older persons’ societal harmony in the Ghanaian context. However, limitations include the reliance on self-reported data and potential generalisability issues. The results of the study should be interpreted with consideration of its methodological constraints aligned with examining associations. This is because the study examined association and therefore it is difficult to establish causality of the dimensions of well-being and the social harmony of older persons in Ghana. Since variables in this study were self-rated by older persons, there is a tendency for respondents to rate their responses in a way considered favourable by others.

## Conclusion

This study shows a multifarious association between dimensions of well-being and the social harmony of older persons. A positive association is found between high levels of emotional and physical well-being and social harmony in older persons. However, older persons with high levels of psychological and spiritual well-being showed less experience of social harmony. Social harmony is influenced by the place of residence of older persons and gender, with those in rural areas and older men linked with higher social harmony than urban dwellers and older women. There is a positive association between formal education and societal harmony, with educated older persons more likely to experience social harmony. Marital status also plays a role, with those who have ever been married (i.e., separated, divorced, or widowed) having higher social harmony. A positive association exists between employment and social harmony of older persons 60 years and older. Lastly, perceived good health decreases the likelihood of social harmony among older persons.

The study has significant implications for policy for improving older persons’ well-being and social harmony. These include addressing the disparities between urban and rural areas, encouraging a safe environment through well-being, gender-sensitive ageing approaches, educational opportunities, support for older adults who are single and do not work, healthcare access, age-friendly communities, community-specific interventions, and psychological and spiritual well-being. Policies and social interventions should consider the various needs and situations of older persons and aim to establish an environment of safety and opportunities for them for higher social harmony in Ghanaian society.
